# An Indwelling Ureteral Stent Forgotten for Over 12 Years

**DOI:** 10.1089/cren.2016.0073

**Published:** 2016-07-01

**Authors:** Samir Bidnur, Melissa Huynh, Nathan Hoag, Ben Chew

**Affiliations:** ^1^ Department of Urological Sciences, University of British Columbia, Vancouver, BC, Canada.; ^2^ Department of Urology, London Health Sciences Centre, London, ON, Canada.

## Abstract

Ureteral stents are one of the most commonly used urologic devices with the purpose of establishing and maintaining ureteral patency. They are also associated with a number of complications including infection, migration, stent-related symptoms, and encrustation, leading to lithiasis. Prolonged stent dwell time is associated with a greater degree of these complications. We present the case of a 36-year-old man who presented with a severely encrusted ureteral stent that had been placed 12.5 years prior for an obstructive left-sided ureteral stone and was lost to follow-up. The patient underwent a combination of percutaneous nephrolithomy, cystolitholapaxy, and ureteroscopy to remove the stent and associated 1.7 cm renal pelvic stone and 4.1 cm bladder stone, necessitating two operative sittings to render him stone free.

## Clinical History

A 36-year-old man presented to the emergency department with several months history of worsening intermittent hematuria, lower urinary tract symptoms, and left flank pain. He had undergone left-sided ureteral stent placement 12 years prior for an obstructive mid-ureteral stone. After stent placement, the patient was referred for shockwave lithotripsy (SWL), but did not return for the procedure and was lost to follow-up. Consequently, the stent was never removed. The patient was otherwise healthy.

## Physical Examination

Physical examination revealed mild suprapubic tenderness, a soft nontender abdomen, and moderate left flank pain. Remainder of the examination was unremarkable.

## Diagnosis

**FIG. 1.**
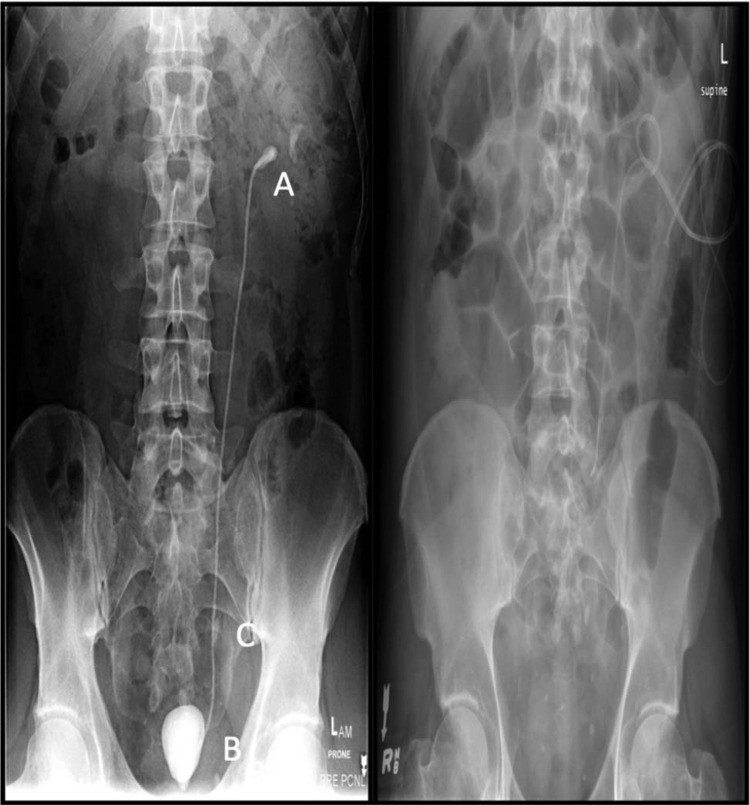
Degree of stone burden associated with 12 years with the indwelling stent. Plain film KUB radiograph shows the proximal and distal calcifications associated with the Double-J stent **(A, B)**, respectively. The original ureteral stone is seen at the level of S2–S3 **(C)**. The patient underwent a combination of left percutaneous nephrolithotomy and cystolitholapaxy to remove the stent and its associated stones. The original left mid-ureteral stone was subsequently treated with URS and laser lithotripsy, after failed shockwave lithotripsy treatment. The patient was left with a stent with a dangle string after the final URS, which was finally removed in outpatient follow–up, leaving him stone and stent free. He remains stone free on follow-up.

A CT–KUB revealed a hydronephrotic left kidney with a retained ureteral stent with major calcifications associated with both distal and proximal pigtails (Fig. 1). The parenchyma of the left kidney was preserved. The proximal pigtail was heavily calcified with a 1.7 cm stone in the renal pelvis, whereas the distal pigtail was profoundly encrusted in a bladder stone measuring 4.1 cm. The length of the stent was mildly encrusted. The original mid-ureteral stone that prompted stent placement 12 years prior was present. The right kidney appeared normal with no evidence of calculi or hydroureteronephrosis.

Blood work, including complete blood count, serum electrolytes, and creatinine, was all within normal limits.

## Intervention

The patient required multiple interventions. Left percutaneous nephrolithomy (PCNL) was performed through lower pole access for proximal stent encrustation. The stone burden was removed under direct nephroscopic observation, and the proximal stent was amputated. The impacted, large bladder stone was fragmented using holmium laser cystolitholapaxy aided by stone crushing forceps. The stent was subsequently removed. Due to prolonged operative time, the patient was brought back for a second operative sitting to treat the original mid-ureteral stone. SWL was initially attempted but was ineffective. The patient then underwent uncomplicated ureteroscopy (URS) with laser lithotripsy, with a stent left with a dangle string.

## Follow-Up

The patient did well postoperatively. Imaging after first operative sitting confirmed only the presence of the original left mid-ureteral stone. SWL was attempted but was ineffective and the patient underwent URS to remove this stone. A stent was placed postoperatively with a dangle string and was pulled on routine follow-up.

## Outcomes

The patient did not have any complications, and he is currently stone free. Stone composition suggested calcium oxalate dihydrate. No metabolic abnormalities were found in office follow-up. Renal function remained in the normal range throughout admission and long-term follow–up, although no renal scan was done to determine split function. On perioperative imaging, however, the ipsilateral kidney had normal parenchymal thickness. This case highlights the complications that can arise from prolonged stent dwell times and the degree of intervention necessary to render a patient stone free. To our knowledge this is the longest recorded stent dwell time described in the literature.

## Discussion

In the absence of clear guidelines for removal of retained stents, this problem has been approached with a variety of treatment modalities. These methods include various combinations of PCNL, URS with laser lithotripsy, cystolitholapaxy, and SWL.^1,2^ Encrustation results from calcium and oxalate precipitation on the stent surface after biofilm formation. Urinary proteins and bacterial products adhere to the stent surface facilitating bacterial adhesion and proliferation, creating a biofilm.^3^ The rate of encrustation is dependent on urinary composition, infection status, and metabolic or congenital abnormalities.^2,4^ This can lead to obstruction and subsequent compromise of renal function. Reported risk factors for encrustation include a history of urolithiasis, infection or biofilm formation, stent material, pregnancy, and duration of stenting.^5–7^ Encrustation can occur within weeks, with more than 75% of patients showing evidence of encrustation after 3 months of polyurethane stent placement.^8^

Standardization of stent encrustation has been described as the FECal system (Forgotten, encrusted, calcified), quantified as grades I–V.^9^ Grades IV and V are associated with stent retention times of more than 2 years and are associated with severe encrustation and stone formation of both proximal and distal pig tails. Grades IV and V encrustation require an average of 1.9–2.7 operative sittings to render a patient stone free.^10^

Several approaches to retained stents have been proposed based on the location and severity of encrustation. The longest duration of continuous indwelling ureteral stent placement previously reported was also 12 years, which necessitated cystolithotomy, URS, and PCNL.^11^ For mildly encrusted stents refractory cystoscopic removal alone, SWL or URS with laser lithotripsy has been recommended by several groups.^2,6,12^ PCNL is the method of choice for severe proximal encrustation, large proximal stone burden, or after other interventions have failed chemolysis with Suby G solution or hemiacidrin is generally discouraged and reserved for extreme cases, and open surgery is typically considered a last resort, especially if imaging or renal function suggests poor renal function.

To avoid the problem of forgotten stents in the first place, some advocate for the use of a stent registry that tracks and reminds clinicians about stents that are overdue for removal.^13^ Meanwhile, degradable stents and novel stent coatings have also been investigated as a strategy to prevent bacterial adherence and subsequent encrustation.^14,15^

## Abbreviations Used

SWL: shockwave lithotripsy
PCNL: percutaneous nephrolithomy
URS: ureteroscopy
